# MRSliceNet: Multi-Scale Recursive Slice and Context Fusion Network for Instance Segmentation of Leaves from Plant Point Clouds

**DOI:** 10.3390/plants14213349

**Published:** 2025-10-31

**Authors:** Shan Liu, Guangshuai Wang, Hongbin Fang, Min Huang, Tengping Jiang, Yongjun Wang

**Affiliations:** 1 State Key Laboratory of Climate System Prediction and Risk Management, Nanjing Normal University, Nanjing 210093, China; 2 Key Laboratory of Poyang Lake Wetland and Watershed Research, Ministry of Education, Jiangxi Normal University, Nanchang 330022, China; 3 Tianjin Key Laboratory of Rail Transit Navigation Positioning and Spatio-Temporal Big Data Technology, Tianjin 300251, China; 4 Xi’an Key Laboratory of Territorial Spatial Information, School of Land Engineering, Chang’an University, Xi’an 710064, China; 5 Jiangsu Center for Collaborative Innovation in Geographical Information Resource Development and Application, Nanjing 210093, China

**Keywords:** deep learning, instance segmentation, leaf segmentation, multi-scale feature fusion, plant phenotyping, point cloud

## Abstract

Plant phenotyping plays a vital role in connecting genotype to environmental adaptability, with important applications in crop breeding and precision agriculture. Traditional leaf measurement methods are laborious and destructive, while modern 3D sensing technologies like LiDAR provide high-resolution point clouds but face challenges in automatic leaf segmentation due to occlusion, geometric similarity, and uneven point density. To address these challenges, we propose MRSliceNet, an end-to-end deep learning framework inspired by human visual cognition. The network integrates three key components: a Multi-scale Recursive Slicing Module (MRSM) for detailed local feature extraction, a Context Fusion Module (CFM) that combines local and global features through attention mechanisms, and an Instance-Aware Clustering Head (IACH) that generates discriminative embeddings for precise instance separation. Extensive experiments on two challenging datasets show that our method establishes new state-of-the-art performance, achieving AP of 55.04%/53.78%, AP_50_ of 65.37%/64.00%, and AP_25_ of 74.68%/73.45% on Dataset A and Dataset B, respectively. The proposed framework not only produces clear boundaries and reliable instance identification but also provides an effective solution for automated plant phenotyping, as evidenced by its successful implementation in real-world agricultural research pipelines.

## 1. Introduction

Plant phenotyping serves as a critical bridge connecting plant genotypes with their environmental adaptability, playing a vital role in crop breeding, precision agriculture, and plant biology research [1,2,3]. Leaves, as the primary organs responsible for photosynthesis and transpiration, possess key morphological traits—such as leaf area, leaf inclination angle, and phyllotaxy—that are essential for assessing plant health, photosynthetic efficiency, and biomass [4]. Critically, these structural traits are not merely descriptive; they are direct determinants of physiological performance. Accurate measurement of leaf area and orientation is fundamental for modeling light interception and canopy-level photosynthesis, while tracking their dynamics reveals plant responses to environmental cues and stresses [5,6,7]. Conventional manual measurement methods are time-consuming, labor-intensive, subjective, and often destructive, posing a significant bottleneck for large-scale phenotyping studies. In recent years, three-dimensional sensing technologies like LiDAR and structure-from-motion (SfM) [8,9,10] have enabled the non-destructive acquisition of high-precision point cloud models of plants, offering unprecedented opportunities for automated, high-throughput leaf phenotyping [11,12,13].

Furthermore, the potential of phenotyping extends beyond structure to physiology. For instance, techniques using RGB imagery have been developed to estimate physiological parameters like chlorophyll content [14,15]. However, as noted in studies such as [16], the accuracy of such 2D methods can be limited by plant 3D structure and occlusion, which complicate pixel-to-biomass relationships and mask leaf-to-leaf heterogeneity [17]. This underscores the promise of integrating precise 3D structural data with spectral imaging: a robust 3D segmentation framework like the one proposed here could provide the necessary spatial context to dramatically improve the resolution and accuracy of physiological trait estimation by enabling per-leaf, rather than whole-plant, analysis.

However, automatically isolating individual leaf instances from point clouds of potted plants remains a highly challenging task in computer vision [18,19,20]. The difficulties primarily stem from: (1) High Density and Severe Occlusion: Leaves are often densely layered and heavily occluded from a single viewpoint, with only fragmented parts visible; (2) Geometric Similarity and Adhesion: Leaves from the same plant tend to share similar size and curvature, and are frequently closely attached with blurred boundaries; (3) Non-Uniform Point Distribution: Limited sensor viewpoints lead to significant variations in point density across different parts of the plant, complicating feature learning [21,22,23]. These challenges make it exceptionally difficult to accurately identify and segment each individual leaf instance directly from the point cloud.

Current point cloud instance segmentation methods can be broadly categorized into two types: proposal-based approaches (such as those adapting the Mask R-CNN framework to point clouds) and proposal-free methods (e.g., those based on discriminative embedding learning) [24]. The former heavily relies on the quality of initially generated 3D bounding box proposals [25]. In densely foliated and highly occluded leaf environments, producing high-quality proposals that tightly enclose individual leaves without overlapping is inherently challenging. Failures in proposal generation often lead directly to segmentation errors. The latter type eliminates the need for proposals but typically depends on learning point feature embeddings followed by clustering to separate instances [26]. These methods are susceptible to interference from local geometric similarities, struggling to distinguish between two adjacent yet separate leaves versus one large continuous leaf. This frequently results in under-segmentation or over-segmentation, with imprecise segmentation boundaries [27].

Inspired by the coarse-to-fine cognitive strategy humans employ when observing dense foliage—where fine local structures such as edges and veins are first used to identify tentative boundaries, followed by the integration of global contextual cues like the overall growth pattern to infer leaf identity and extent—we propose the MRSliceNet framework. We recognize that fine-grained local geometry is essential for distinguishing leaf boundaries, while global context is critical for accurately determining leaf affiliation and avoiding misjudgment. Accordingly, the core contribution of this paper is the design of an end-to-end network, with its innovation embodied in three tightly integrated modules:

- Multi-scale Recursive Slicing Module (MRSM): This module simulates the process of “examining” local structures from multiple perspectives. It recursively slices the point cloud using multiple orthogonal planes across multi-scale spaces and employs a lightweight sub-network to extract rich local geometric features from each slice. This strategy efficiently and focusedly captures details of key regions such as leaf margins and tips.
- Context Fusion Module (CFM): To integrate the multi-scale local features extracted by MRSM with global morphological information, the CFM module aggregates features across different scales and locations via attention mechanisms or graph convolutional networks. This enriches the feature representation of each point with both its local structural context and its position within the global structure, effectively addressing occlusion and adhesion issues.
- Instance-Aware Clustering Head (IACH): The network ultimately outputs instance-aware feature embeddings for each point through this clustering head. Unlike simple clustering, this module is guided by a loss function that ensures points from the same instance are highly compact in the feature space, while those from different instances are distinctly separated, enabling accurate and automatic instance segmentation.

To validate the effectiveness of MRSliceNet, we hypothesized that its integrated design—combining multi-scale recursive slicing, context fusion, and instance-aware clustering—would achieve superior segmentation performance by more effectively handling occlusion, geometric similarity, and uneven point density compared to existing state-of-the-art methods. We tested this hypothesis through extensive experiments on two challenging datasets. The proposed framework demonstrates strong potential for integration into automated phenotyping pipelines, and future work will focus on its deployment and longitudinal evaluation in real-world agricultural and breeding scenarios.

## 2. Related Work

Point clouds are more promising for plant phenotyping analysis than optical images because they present accurate three-dimensional structural and spatial information. In addition, point clouds are robust to occlusion and illumination variations; they have become the primary data source for quantifying plant architectural traits, especially in dense canopies. Owing to the accessibility of 3D scanning technologies, traditional supervised machine learning methods are commonly used for organ-level segmentation [28]. The performance of these methods relies on two important factors: distinctive geometric features and discriminative classifiers. Li et al. [29] extracted key components of wheat plants by incorporating shape descriptors, geometrical properties, and spatial context. This approach was used to separate ears from stems and leaves. Zhang et al. [30] identified different plant organs from 3-D point clouds according to a combination of geometric and normal-based features. These methods focus heavily on geometry and spatial relationships; however, recognizing organs from plant point clouds based solely on a geometrical point of view is often insufficient for complex architectures.

Advancements in deep learning methods for point cloud segmentation have led to growth in plant phenotyping [31]. PointNet and DGCNN were combined by DeepSeg3DMaize [32] to achieve performance comparable to traditional methods in a leaf-stem segmentation study. The main drawback is that it has only been implemented on maize plants; therefore, it is difficult to determine whether the algorithm is robust enough to apply to different crop species. SPTNet integrates Sparse Convolution (SpConv) and Transformer networks for leaf-wood separation [33]. The SpConv blocks enable efficient local feature extraction, while the Transformer addresses the limitations of global feature extraction in SpConv blocks. The point feature extraction block is specifically designed to assist the segmentation task, and each geometric feature block implements distinct adaptive radius strategies to reduce reliance on prior knowledge. Genze et al. [34] developed a UNet-like architecture that could adapt to the morphological differences among individual leaves, thereby achieving a good understanding of the complex weed structure. In addition, there are methods based on deep learning for extracting instances from plant point clouds.

Point cloud instance segmentation aims to annotate every point with both a semantic label and an instance identifier, which is crucial for fine-grained scene understanding in various applications [35]. Specifically, point cloud instance segmentation techniques significantly advance plant phenotyping by enabling the automated, precise extraction of organ-level traits. These methods successfully tackle the challenges of dense foliage and complex plant architectures, allowing for the accurate identification, separation, and counting of individual organs like leaves and stems [36]. This capability unlocks high-throughput quantification of previously difficult-to-measure traits, such as individual leaf area, stem diameter, and 3D leaf angle distribution, thereby providing deeper insights into plant growth and function.

Leaf instance segmentation is among the most challenging tasks in plant phenotyping [37]. To avoid the interference of mutual leaf occlusion, Jiang et al. [38] divided plant point clouds into superpoint regions and developed an instance segmentation system for roadside trees. Measuring leaf angle distribution at the organ level is also important. Li et al. [39] identified the contextual features of plant point clouds using graph convolutional networks and then input them into a segmentation network to recognize individual leaf instances. To improve the accuracy of leaf counting, Du et al. [40] developed a novel transformer-based approach that combines a convolutional neural network and a mean-shift clustering algorithm. The inherent geometric information hidden in point clouds was investigated by Mirande et al. [41] by introducing an edge-informative graph to describe adjacency relationships between the neighboring regions. In addition, they designed a new graph convolutional network for deep feature extraction, obtaining the final leaf instances by semantically annotating points at different granularity levels. To extract multiple organs from dense plant point clouds, Yang et al. [42] constructed an encoder–decoder model. An efficient feature encoder can repair detailed information and contribute to feature learning for predictions. The attention mechanism is becoming increasingly important because it can enhance discriminate information and suppress interference. In TrackPlant3D [43], a dual attention module, together with a weighted cross-entropy loss, was developed to integrate local information with global dependencies. TrackPlant3D can perform multiclass semantic segmentation of different organs in plant point clouds. For the semantic segmentation of large-scale field-based plant point clouds, an attention-aggregation network was proposed to integrate neighboring features of all points in PhenoNet [44].

## 3. Materials and Methods

**Figure 1:**
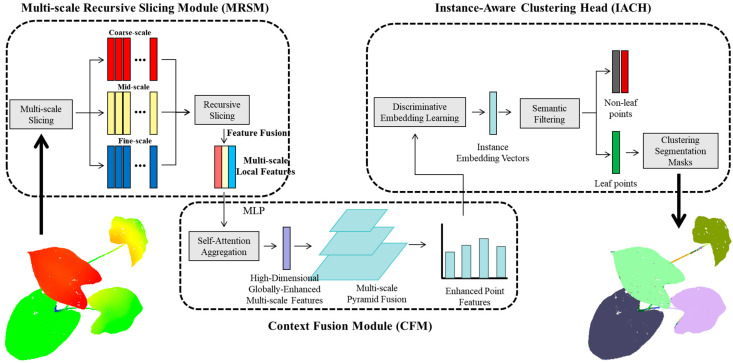
Workflow of the MRSliceNet framework for vegetation point cloud segmentation. The diagram illustrates the step-by-step processing pipeline: starting with Multi-scale Slicing to capture structural features at different levels, followed by Recursive Slicing for fine-grained partitioning. Discriminative Embedding Learning then converts these slices into feature vectors, which are refined through Semantic Filtering to remove noise. The process culminates in Clustering Segmentation to group points into distinct vegetation components, aided by Self-Attention Aggregation that leverages contextual relationships (as indicated by the arrows directing flow and information integration between steps) to enhance segmentation accuracy.

This paper presents a novel method for leaf instance segmentation in plant point clouds. Our study utilized point cloud data from plants at a consistent developmental stage (e.g., mature vegetative stage) to minimize variability in leaf size and complexity. The proposed approach introduces a Multi-scale Recursive Slicing Module (MRSM) to extract discriminative local geometric features by recursively slicing the point cloud at various scales, capturing details from overall leaf orientation to fine margins and venation. Subsequently, a Context Fusion Module (CFM) integrates these multi-scale, fragmented features using a self-attention mechanism and feature pyramid fusion, enriching each point’s representation with global semantic context. Finally, an Instance-Aware Clustering Head (IACH) learns a discriminative embedding space where points of the same leaf cluster together and those of different leaves are separated, enabling accurate instance segmentation via offline clustering, enhanced by semantic filtering to exclude non-leaf points. This design aims to achieve robust segmentation despite potential minor morphological variations inherent even among the same-stage plants. The overall workflow is illustrated in Figure 1.

### 3.1. Discriminative Feature Extractor for Leaf Segmentation in Plant Point Clouds

**Figure 2:**
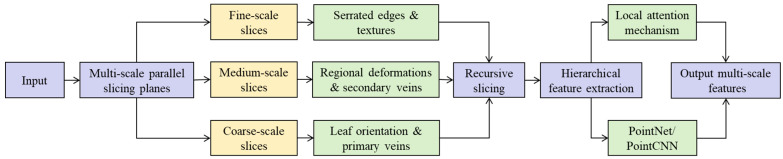
The overview of the Multi-scale Recursive Slicing Module. The diagram depicts the feature extraction process from an input point cloud. The cloud is processed through multi-scale parallel slicing planes to generate coarse-scale (capturing leaf orientation and primary veins) and medium-scale (capturing regional deformations and secondary veins) slices. These slices then undergo recursive slicing and hierarchical feature extraction via PointNet/PointCNN (as indicated by the arrows), ultimately yielding a comprehensive set of multi-scale features.

In the context of plant point cloud processing, particularly for leaf segmentation and classification, learning features directly from raw, unordered point clouds often faces a key challenge: fine local details—such as serrated leaf margins, venation patterns, and surface texture—are frequently overwhelmed by the overall canopy structure or interference from adjacent leaves. To address this issue, the MRSM is proposed as shown in Figure 2. Its core idea is to recursively slice the point cloud using planes across multiple scales and hierarchical depths, thereby systematically “dissecting” complex plant structures to maximize the exposure and capture of discriminative local geometric features—including leaf edge contours, curvature variations, primary and secondary vein ridges, and subtle deformations. This approach is particularly designed for and evaluated on point clouds representing the entirety of the plant’s above-ground architecture, ensuring that features are extracted cohesively across all regions of the canopy, from the dense top to the sparse bottom. This significantly enhances the boundary accuracy and structural integrity of leaf segmentation.

#### 3.1.1. Multi-Scale Slicing Strategy

Moving beyond conventional methods that rely on a single fixed slice thickness, our approach adaptively slices the input vegetation point cloud using a set of parallel slicing planes at multiple, collaboratively designed scales (coarse, medium, and fine). The slicing intervals at these scales are not fixed but are determined relative to the morphological hierarchy of the plant and the spatial resolution of the point cloud [45]. Specifically:

(1) The coarse-scale (with larger intervals, typically 5–10% of the plant height) captures the overall architecture, such as primary branch orientation and the global distribution of leaves, while being robust to noise and fine-scale clutter.
(2) The medium-scale (with intervals calibrated to the size of typical leaf clusters or individual leaves) reveals meso-structural patterns like regional leaf deformations and secondary venation.
(3) The fine-scale (with smaller intervals, approaching the resolution limit of the point cloud) precisely extracts high-resolution details, including serrated leaf margins, subtle curls, and insect perforations.

This self-adaptive, multi-scale strategy ensures that our method is robust across diverse tree species, growth stages, and organ sizes, as it dynamically adjusts to variations in canopy density, occlusion, and point cloud quality, rather than relying on species-specific parameter tuning.

#### 3.1.2. Recursive Operation Mechanism

At each selected scale, the slicing operation is performed recursively in a layered manner. Specifically, for each sub-region generated from the previous slicing step, a new slicing direction and thickness are automatically determined based on its internal spatial distribution characteristics (e.g., point density and normal variation) to initiate a new round of slicing. This recursive process is controlled by a set of termination criteria to ensure efficiency and prevent over-segmentation. Point count threshold: Recursion stops when a sub-region contains fewer than a predefined number of points, ensuring statistical significance; Geometric simplicity criterion: Recursion terminates if a sub-region’s geometry becomes sufficiently simple, as measured by the variance of point normals or dimensionality features; Maximum depth limit: A safety cap on the recursion depth is enforced to align with the natural hierarchical levels of plant structures.

Governed by these criteria, the recursion enables the network to progressively focus on more localized and discriminative regions, analogous to a controlled “zooming-in” process [46]. This approach allows the module to thoroughly explore fine-grained geometric patterns within and along the edges of leaves while preserving their global contextual connection. More importantly, by being constrained by data-inherent properties rather than task-specific optimization, this recursive mechanism enhances feature discriminability without overfitting to training-set noise, significantly improving the model’s ability to generalize across varying degrees of leaf overlap and adhesion.

#### 3.1.3. Feature Learning and Fusion

For each “point cloud slice” obtained from a slicing operation—i.e., the set of points lying between two adjacent slicing planes—we employ a lightweight feature encoding subnetwork (such as a variant based on PointNet or PointCNN) to learn deep features of the local segment. These features encapsulate not only geometric attributes (e.g., coordinates, normals, curvature) but also spatial distribution relationships and statistical properties within the slice. To further enhance feature discriminability, a local attention mechanism is introduced to amplify feature responses in key regions such as leaf edges and vein structures.

#### 3.1.4. Output and Feature Enhancement

Multi-scale local features learned across all recursive slicing paths are aggregated through a backtracking mechanism and assigned to each point in the original point cloud. As a result, every point obtains a set of enhanced feature vectors rich in multi-scale local contextual information, which are highly sensitive to discriminative regions such as leaf edges, veins, and surfaces. The final point features integrate structural information spanning from macroscopic to microscopic levels, effectively supporting subsequent point cloud segmentation networks in achieving more precise leaf instance segmentation and semantic labeling. This significantly improves the accuracy and reliability of vegetation point cloud analysis.

### 3.2. Integration and Enhancement of Cross-Slice Semantic Information

The MRSM successfully captures rich local geometric features of the point cloud from multiple scales and perspectives through parallel and heterogeneous slicing paths. While these features are highly detailed, they are inherently fragmented, heterogeneous, and observed from multiple localized viewpoints. They can be thought of as close-up photos taken by multiple cameras from different angles—each captures fine details but lacks global contextual coherence. If these features are directly used for final leaf instance segmentation, particularly in complex, dense, or severely occluded potted plant environments, the model may mistakenly group points from different leaves into the same instance.

**Figure 3:**
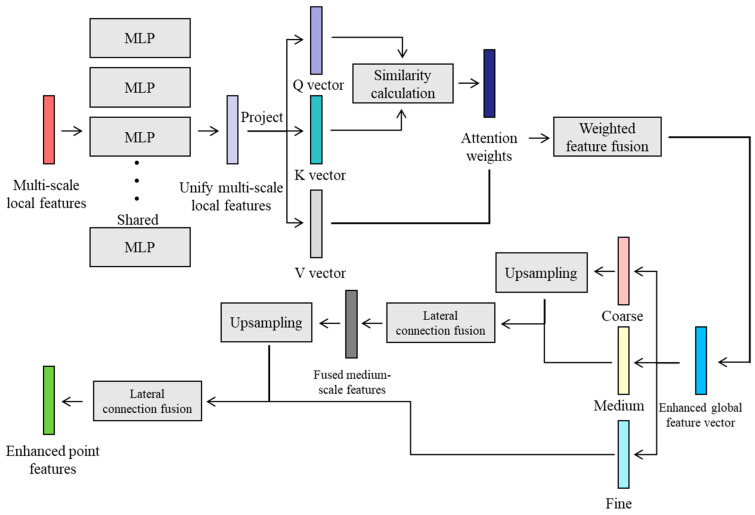
The architecture of the Context Fusion Module for multi-level feature integration. The module receives inputs from multiple layers (depicted as parallel MLP blocks). It first computes feature similarities, then performs weighted fusion based on these similarities. The fused features are upsampled and further integrated with preceding features via lateral connections, ultimately generating refined, context-aware feature maps.

As shown in Figure 3, the CFM is designed to function as a powerful “information hub” or “global scheduling center”. Its core objectives address three critical challenges: (1) Integration: how to align and fuse heterogeneous features from different slicing paths within a unified semantic space; (2) Enhancement: how to inject global semantic context into the local features of each point; and (3) Consistency: how to ensure the resulting point feature representations preserve both the precision of local details and coherence with the overall plant structural semantics [47]. Through the processing of the CFM, each point acquires a novel, powerful feature representation—a function of both its local neighborhood information and its global position within the overall point cloud—thereby providing highly discriminative input for the subsequent instance segmentation head.

#### 3.2.1. Feature Alignment and Aggregation for Global Context Encoding

The multiple sets of features originating from the MRSM module first face an “alignment” problem. These features are derived from different slicing planes (which may have different normal vectors) and different network branches (which may have varying receptive fields and feature dimensions), making direct fusion challenging and inefficient. We first map all input feature vectors into a unified high-dimensional semantic space (e.g., 256 or 512 dimensions) using a shared Multi-Layer Perceptron (MLP) [48]. This mapping process not only unifies their dimensionality but also serves as a semantic alignment step. Through nonlinear transformations, geometrically diverse cues extracted from different paths—such as contour information from the XY-plane and curvature details from normal vector space—are converted into comparable vector representations with a common semantic foundation.

The aligned features form a feature matrix F of the point set. Subsequently, we employ a Self-Attention-based mechanism [49] to achieve global information aggregation across slices and points. Due to its powerful capacity for modeling long-range dependencies, the self-attention mechanism naturally serves as the preferred tool for implementing this “information hub” function. The computational process can be formulated as follows: (1) *Linear Projection*. For each point *i* with feature $f_{i}$, we compute its query vector $q_{i}=W_{Q}\times f_{i}$, key vector $k_{i}=W_{K}\times f_{i}$, and value vector $v_{i}=W_{V}\times f_{i}$ using learnable weight matrices $W_{Q}$, $W_{K}$, and $W_{V}$, respectively. (2) *Attention Weight Calculation*. The attention weight $a_{i,j}$ (see Equation (1)) measures the importance of point *j*’s features to point *i*. This is computed by taking the dot product between $q_{i}$ and the key vector $k_{i}$ of every point *j*, followed by normalization via a Softmax function [50].

(1) $$a_{i,j}=\frac{\;exp(\frac{q_{i}\cdot \;k_{j}^{T}}{\sqrt{d}}\;)\;}{\sum_{k=1}^{N}exp(\frac{q_{i}\cdot \;k_{j}^{T}}{\sqrt{d}}\;)},$$

where *d* denotes the dimension of the query/key vectors, and the division by $\sqrt{d}$ serves as a scaling factor to prevent excessively large inner products that may lead to vanishing gradients.

(3) *Weighted Summation*: The updated feature $f_{i}^{\prime }$ (see Equation (2)) for point *i* is computed as the weighted sum of the value vectors $v_{j}$ from all points, using the attention weights $a_{i,j}$ obtained in the previous step.

(2) $$f_{i}^{\prime }=\sum_{j=1}^{N}a_{i,j}\cdot v_{j},$$

The strength of this mechanism lies in its global nature and adaptability. Thanks to its global receptive field, each point can directly access and integrate feature information from all other points—irrespective of their original slicing paths. For instance, a point on an occluded leaf can effectively “query” visible points that represent the main structure of the leaf through attention, enabling it to infer its own semantic identity accurately. Moreover, the model demonstrates high adaptability by automatically learning attention weights in a data-driven manner, without relying on handcrafted rules. When determining the leaf affiliation of a point, the model adaptively focuses on: (1) neighboring points within the same slice to enforce local geometric consistency, (2) points from different slices but belonging to the same leaf to incorporate cross-view validation, and (3) points on the stem or main branches to leverage global structural context for distinguishing between adjacent leaf clusters. Through the self-attention layer, each point acquires an enhanced feature representation enriched with global context from the entire point cloud. To further improve representational capacity and training stability, we typically stack multiple such self-attention blocks, incorporating residual connections and layer normalization to form a standard Transformer encoder architecture.

#### 3.2.2. Integrating Multiscale Features via Pyramidal Fusion

Although the self-attention mechanism implicitly processes information at different scales, explicitly incorporating multi-scale fusion has been proven highly effective for dense point cloud segmentation tasks. The MRSM inherently provides multi-scale features extracted from slicing paths at various resolutions. In the CFM, inspired by the Feature Pyramid Network (FPN) [51], we construct a lightweight point cloud feature pyramid that integrates these multi-scale features through top-down and lateral connections.

We treat the features output by branches at different scales in the MRSM as distinct levels of a pyramid. For instance, features from the slicing path with the smallest receptive field correspond to Level 0 (high resolution, rich in detail), while those from the path with the largest receptive field form Level 2 (low resolution, semantically stronger). Starting from the features at the coarsest scale (Level 2), we first upsample its feature map—typically using trilinear interpolation or nearest-neighbor interpolation—to match the spatial resolution (i.e., point density) of the next finer scale (Level 1). The upsampled coarse-scale features are fused with the fine-scale features from the corresponding scale of the MRSM. Since both have already been semantically aligned through the preceding self-attention module, we can directly employ element-wise addition or channel concatenation followed by a small MLP for fusion. This step is critical: the coarse-scale features provide strong semantic cues about “what it is” (e.g., this is a long, curved leaf), while the fine-scale features offer precise positional information about “where the edges are”. The fused Level 1 features are further upsampled and undergo the same lateral connection and fusion operation with the Level 0 features. Finally, we obtain a set of fused multi-scale feature maps. Typically, fused features from all levels are extracted and aggregated into the final point features via skip connections, or the output from the most detail-rich fused level (Level 0) is selected as the primary output.

Overall, the pyramid fusion mechanism brings substantial benefits to leaf segmentation: (1) Addressing Adhesion Issues. In cases of large-area leaf adhesion, boundaries can appear highly ambiguous or even intertwined at fine scales. However, at coarse scales, their overall orientation (main vein direction), curvature, and connection points to the stem may differ significantly. Pyramid fusion allows the features at each point to incorporate information from both perspectives, enabling the classifier to clearly distinguish between adhered boundaries. (2) Improved Robustness. Leaves may be partially occluded, damaged, or exhibit abnormal morphology. While fine-scale features can become unreliable under such conditions, coarse-scale structural characteristics remain more stable. The fused features, thus, exhibit stronger robustness against these types of noise. (3) Detail Preservation. Compared to using coarse features alone, the fusion process preserves fine geometric details in critical areas such as leaf margins and tips, ensuring high precision in the segmentation boundaries.

#### 3.2.3. Highly Descriptive Point Features

After a series of processing steps, including feature alignment, global self-attention aggregation, and multi-scale pyramid fusion, the CFM module outputs a new high-dimensional feature vector for each point in the input point cloud. The semantic richness of these feature vectors is fundamentally enhanced: (1) They are not only aware of “what the local geometric structure around me (this point) is” (provided by the MRSM module); (2) But also understand “where this local structure is located within the entire plant and what role it plays” (incorporated by the CFM module). For example, a point located on the periphery of the point cloud with high curvature might be initially recognized by the MRSM as “possibly the edge of a leaf.” The CFM module then enriches this point with contextual awareness: “the leaf you belong to is drooping downward and is far from the main stem; the adjacent high-curvature point near you is actually part of a completely different plant.” Such local features with global understanding significantly enhance the model’s discriminative capability in complex scenarios.

Finally, these enhanced point features from the CFM module are fed into a lightweight, parallel segmentation head (typically a shared MLP). This head simultaneously performs two tasks: (1) Semantic Segmentation: predicting the class probability for each point among “background”, “stem”, or “leaf”; (2) Instance Segmentation: typically achieved either by predicting vector offsets between points and their instance centers to cluster points into respective instances, or by directly learning a per-point instance embedding followed by a clustering method (such as mean shift) to distinguish individual leaf instances. Thanks to the powerful features provided by the CFM module, this segmentation head can accomplish its tasks with high accuracy. Even when processing highly dense and adherent point clouds of potted plants, it clearly and precisely separates each individual leaf, laying a solid foundation for subsequent plant phenotyping analysis. In summary, by cleverly integrating self-attention mechanisms and a feature pyramid structure, the CFM module successfully consolidates and enhances semantic information across slices and scales. It elevates fragmented local observations into a coherent global understanding, serving as the indispensable “brain” of the entire point cloud instance segmentation architecture.

### 3.3. Instance-Aware Clustering Head for Discriminating and Segmenting Leaves

**Figure 4:**
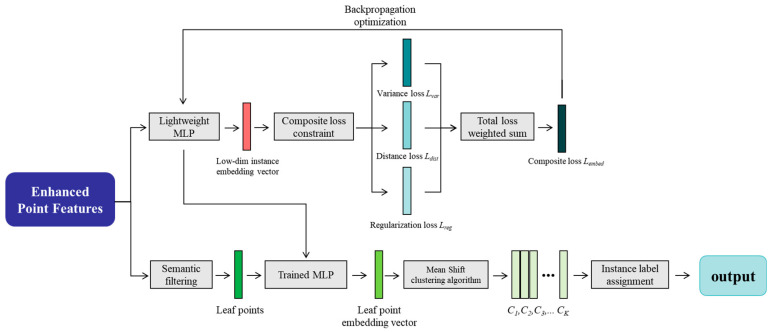
Workflow of the Instance-Aware Clustering Head for point cloud instance segmentation. The process begins with enhanced point features that are refined through a semantic filtering step. These filtered features are processed by a lightweight MLP, which is trained under a composite loss constraint (with the total loss being a weighted sum of individual components). The output features from the trained MLP are then fed into the Mean Shift clustering algorithm to group points into distinct instances, culminating in the final instance label assignment for each point.

After deep processing by the CFM module, each point in the point cloud has acquired a powerful feature representation that integrates both local geometric details and global semantic context. However, these features alone cannot directly produce the final instance segmentation result—that is, explicitly assigning each point to a specific leaf. This final and critical step is accomplished by the IACH. As shown in Figure 4, the core idea of IACH is to transform the instance segmentation problem into a metric learning task in feature space. By guiding the model to learn an embedding space that follows the principle of “pull points of the same instance together, push points of different instances apart,” it elegantly addresses the challenge of segmenting highly irregular and severely adherent leaves.

#### 3.3.1. Towards a Discriminative Instance Embedding Space

Traditional instance segmentation methods (e.g., Mask R-CNN) heavily rely on bounding boxes as initial detection results [52]. However, this paradigm faces fundamental limitations in the context of segmenting leaves in dense potted plant point clouds. (1) Spatial Inefficiency: Leaves are extremely thin, curved, surface structures in 3D space. Over 90% of the volume within a tightly fitted 3D axis-aligned bounding box used to enclose a leaf is occupied by empty space, while the remaining portion is inevitably contaminated by points from other leaves or stems, resulting in significant confusion. (2) Severe Overlap: In potted plants, leaves are often densely layered, leading to extensive overlap between their 3D bounding boxes. This makes post-processing steps like Non-Maximum Suppression (NMS) [53] less effective and may even erroneously suppress valid leaf instances. (3) Shape Misalignment: Rectangular bounding boxes are inherently mismatched with the irregular shapes of natural leaves, making it difficult to provide accurate initial localization. Based on this, the bounding box approach is entirely abandoned in our IACH module, and a paradigm of Discriminative Embedding Learning is adopted instead. This method operates without relying on a detect-then-segment pipeline. Instead, each point is assigned a feature representation such that instance membership is implicitly and naturally expressed by the relative distances between points in the embedding space. This approach is particularly well-suited for objects like leaves, which have no prior shape, are highly irregular, and are densely distributed.

The IACH itself is implemented as a lightweight neural network, typically composed of several fully connected (MLP) layers. The high-dimensional point features $f_{i}$ output by the CFM module are taken as input, and a lower-dimensional (e.g., 32 or 64-dimensional) instance embedding vector $e_{i}$ is produced for each point. The learning objective for this embedding vector is clearly defined: in the learned embedding space, all points belonging to the same leaf should be closely clustered together, while points from different leaves are pushed apart, maintaining sufficient distance from each other.

To achieve this objective, a specially designed loss function is required to drive the model toward learning this “intra-cluster cohesion and inter-cluster repulsion” property. Typically, a composite loss function is employed, which integrates multiple constraints.

(1) Variance Loss (see Equation (3)): The distance between points within the same instance cluster and their cluster center (mean) is constrained by this term. For a given leaf instance S containing N points, the cluster center of their embedding vectors is defined as $\mu_{S}=\;(1/N)\;\times \;\sum_{i\in S}e_{i}$. All points within the cluster are required to maintain a distance to $\mu_{S}$ that is less than a predefined threshold $\delta_{v}$.

(3) $$L_{var}=\frac{1}{C}\times \sum_{S}\left[\frac{1}{N_{S}}\sum_{i\in S}{\left[\left\|\mu_{S}-e_{i}\right\|-\delta_{v}\right]}_{+}^{2}\right],$$

where *C* is defined as the number of instances within the batch. The hinge function, denoted as [·]₊ (e.g., ReLU), is applied so that a penalty is imposed only when the distance exceeds the threshold $\delta_{v}$. By minimizing $L_{var}$, the model adjusts the feature representations $e_{i}$ of the samples, pulling all samples as close as possible into a “circle” centered at their respective cluster center with a radius of $\delta_{v}$. All samples falling within the circle (distance ≤ $\delta_{v}$) are considered “safe” and remain unpenalized (with zero loss). Any sample located outside the circle (distance > $\delta_{v}$) will be pulled toward the circle. The farther it is from the circle, the stronger the pulling force becomes (due to the squared term causing a rapid increase in loss).

(2) Distance Loss (see Equation (4)): The separation between the centers of different instance clusters is constrained by this term. For two distinct cluster centers, $\mu_{S}$ and $\mu_{T}$, their distance is required to be greater than a predefined threshold $\delta_{d}$ (where $\delta_{d}>\delta_{v}$).

(4) $$L_{dist}=\frac{2}{C(C-1)}\times \sum_{S=1}^{C}\sum_{T=1,\;T\neq S}^{C}{\left[\delta_{d}-\left\|\mu_{S}-\mu_{T}\right\|\right]}_{+}^{2},$$

This loss term is designed to penalize cluster centers that are positioned too closely together, thereby forcing the embeddings of different instances to be pushed apart by the network.

(3) Discriminative Loss (Regularization Term, see Equation (5)): To ensure training stability and prevent collapse of the embedding space (where all points converge to the same location), a regularization term is typically introduced. This term is designed to pull all cluster centers toward the origin, or alternatively, more sophisticated metric learning losses such as Triplet Loss may be employed. A commonly adopted regularization term in this work is defined as:

(5) $$L_{reg}=\;\frac{1}{C}\times \sum_{S=1}^{C}\left\|\mu_{S}\right\|,$$

This prevents the norms of the embedding vectors from increasing without bound.

The final training loss is defined as a weighted sum of the aforementioned terms:

(6) $$L_{embed}=\alpha L_{var}+\beta L_{dist}+\gamma L_{reg},$$

By minimizing $L_{embed}$, the IACH module is trained to function as an efficient encoder, through which the powerful semantic-geometric features provided by the CFM are mapped into a well-structured metric space. In this space, instance discrimination is no longer dependent on ill-defined boundaries, but is instead determined by straightforward Euclidean distances between points. The hyperparameters α, β, and γ are weighting coefficients that balance the contribution of these three loss terms. Their values were determined through an empirical search on the validation set. The goal of this search was to achieve stable training dynamics and optimal segmentation performance, ultimately leading to the final set of values (α = 0.5, β = 0.3, γ = 0.2). This combination effectively ensured that none of the loss terms dominated the gradient during optimization, allowing the model to learn a well-structured embedding space where instances are both compact and well-separated.

#### 3.3.2. From Embedding Space to Segmentation Masks

During the inference phase, which begins after model training is completed, the formation of clusters is no longer guided by ground truth labels. The entire point cloud is fed into the network, where it is processed sequentially by the MRSM and CFM modules. Finally, an instance embedding vector $e_{i}$ is predicted for each point by the IACH module. The point embedding vectors $\left\{e_{i}\right\}$ are fed into a simple and computationally efficient offline clustering algorithm. Due to the optimization of the loss function during the training phase, a well-separated, instance-oriented cluster structure has been formed in the embedding space. Each cluster $C_{k}$ output by the clustering algorithm directly corresponds to a leaf instance in the point cloud. A unique instance label *k* is assigned to every point within cluster $C_{k}$.

Despite the strong discriminative power of the embedding learning process itself, stem points may be positioned in close proximity to leaf points in extremely dense scenes or those with complex stem structures, occasionally leading to clustering errors. To further enhance segmentation accuracy and robustness, a lightweight semantic filtering step is introduced. This step is typically performed prior to clustering. A lightweight semantic segmentation head (also a small MLP) is configured in parallel, which is fed with the same CFM features and used to output the semantic class probability (e.g., “stem”, “leaf”) for each point. This head is trained with a standard cross-entropy loss function and optimized simultaneously with the main embedding learning task. During inference, the semantic prediction is first obtained for each point. Subsequent instance embedding clustering is applied only to those points classified as “leaf”. Points categorized as “stem” or “background” are directly excluded from the instance clustering process.

This strategy provides two significant benefits. First, potential interference from stem points is completely eliminated from the leaf instance clustering process, allowing the clustering algorithm to focus solely on distinguishing between different leaves [54], thereby simplifying and purifying the task. Second, the number of points that need to be clustered is greatly reduced, which decreases the computational overhead of the clustering algorithm and accelerates the overall inference speed. In summary, through the combination of discriminative embedding learning and efficient clustering algorithms, enhanced by semantic filtering, the Instance-Aware Clustering Head (IACH) successfully achieves the final objective of transforming powerful point features into precise instance segmentation results. It is well-suited to the irregular, dense, and adherent nature of leaves in natural scenes, providing a reliable technical endpoint for automated plant phenotyping analysis.

### 3.4. Implementation Details

The proposed neural network was implemented using the PyTorch framework (version 2.0.1). All experiments were conducted on a workstation running the Ubuntu 20.04 LTS operating system. The hardware configuration included an Intel Xeon Silver 4210 CPU @ 2.20GHz, 64 GB of DDR4 RAM, and an NVIDIA GeForce RTX 3090 GPU with 24 GB of VRAM [55]. The core software environment was Python 3.9, and the model relied on key libraries including CUDA 11.8 and cuDNN 8.6 for GPU acceleration, NumPy (1.24.0) for numerical operations, and SciPy (1.10.0) for scientific computing.

The model was trained using the Adam optimizer with an initial learning rate of 0.001 and a batch size of 8. We trained the model for 150 epochs, which was sufficient for the training loss to converge and plateau. The total training time on the primary dataset [56] was approximately 18 h. For inference, the average processing time per plant point cloud was around 0.5 s, demonstrating the efficiency of our method for practical applications.

## 4. Results

### 4.1. Dataset Description and Evaluation Criteria

Experiments were conducted using point cloud data of potted plants collected from multiple sources to ensure diversity and robustness. For training the neural network, we utilized the publicly available, manually annotated dataset introduced by [56]. This dataset comprises point clouds of 500 individual potted plant specimens, encompassing a wide variety of species with significant morphological differences (e.g., variations in leaf size, shape, and plant architecture). The training set includes common ornamental species such as *Ficus benjamina*, *Dracaena fragrans*, and *Hedera helix*, covering a range of plant architectures from shrubs to small trees.

**Figure 5:**
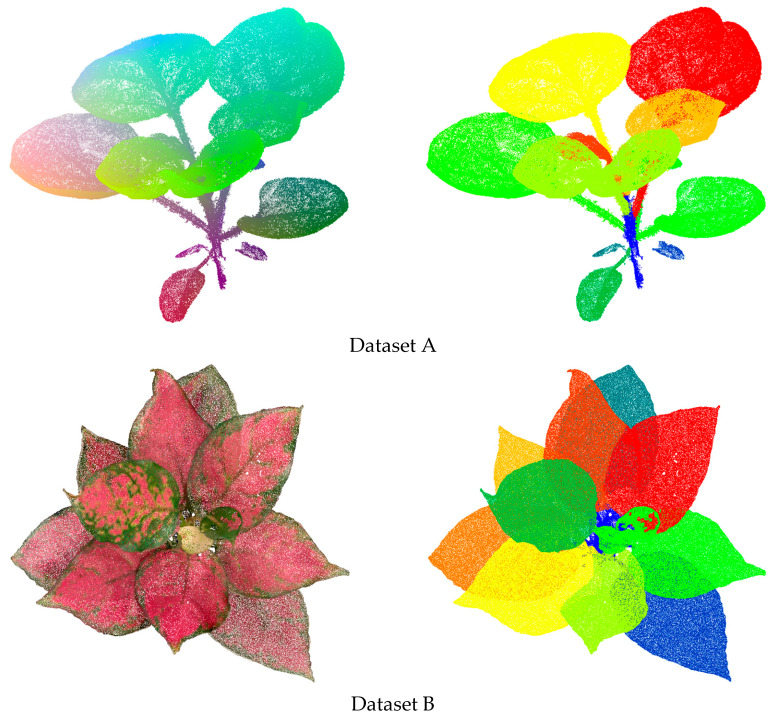
Examples of the manual segmentation of the plant point clouds in the two benchmarks. In the left panel, the original data are displayed: the coloration in Dataset A corresponds to height values, and the coloration in Dataset B corresponds to textural properties. The right panel presents the instance-wise annotations, with each distinct color denoting a separate instance.

To rigorously validate the proposed method and assess its generalizability, we employed two independent datasets derived from distinct botanical collections, as illustrated in Figure 5. The first validation set (Dataset A) is a LiDAR point cloud dataset containing 350 potted plant samples acquired using a 3D laser scanner. This collection includes species with distinct leaf morphologies, such as the broad, simple leaves of *Philodendron hederaceum* and the finely dissected leaves of *Schefflera arboricola*, representing young to mature growth stages. From this set, 130 samples were manually annotated to serve as ground truth for quantitative evaluation. The second validation set (Dataset B) is an image-based point cloud dataset comprising 320 samples reconstructed from multi-view images captured with a Canon EOS 5D Mark IV camera, featuring species like *Monstera deliciosa* and *Ficus lyrata,* which exhibit complex leaf geometries and natural occlusion patterns. Detailed instance-level annotations available for 130 specimens.

The plant samples across all datasets were not randomly sampled from a single population but were systematically selected from different sources to maximize the coverage of biological variation. This strategy, which includes a high number of specimens, a diverse range of species, and different data acquisition modalities (LiDAR vs. image-based), is designed to rigorously test and support the generalization of our method to a broad spectrum of potted plants.

In the performance evaluation of instance segmentation for potted plant point clouds, the following Average Precision (AP)-based metrics are adopted to systematically measure the multi-scale recognition capability of the model under different IoU thresholds [57,58,59]: (1) AP. The mean average precision of leaf instance segmentation is calculated over IoU thresholds ranging from 0.5 to 0.95 with a step size of 0.05, providing a comprehensive measure of segmentation consistency across various levels of strictness. (2) AP50. With the IoU threshold set to 0.5, this metric computes the average precision under a relaxed matching criterion, reflecting the model’s detection performance when larger boundary deviations are tolerated. (3) AP25. AP25 is defined with an IoU threshold of 0.25, through which instance recognition performance is evaluated under significant boundary tolerance. This metric is designed to be suitable for sensitivity analysis of initial localization or coarse segmentation results.

This evaluation framework focuses on segmentation quality across different IoU strictness levels, providing a comprehensive assessment of instance-level recognition performance from lenient to stringent matching conditions [60]. It is particularly well-suited for potted plant point cloud scenarios where leaves exhibit complex morphology and severe mutual occlusion.

### 4.2. Qualitative Results

**Figure 6:**
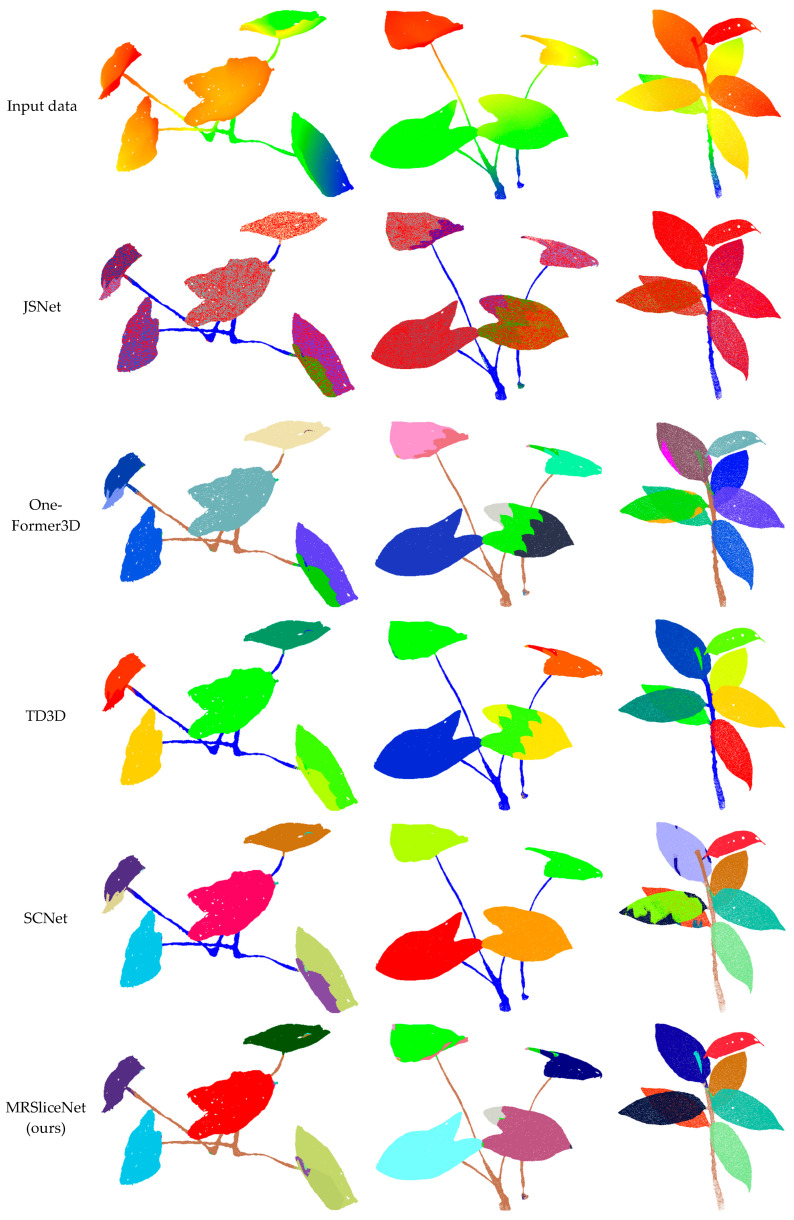
Segmentation results of the Dataset A plant point cloud. Different colors represent different instances.

**Figure 7:**
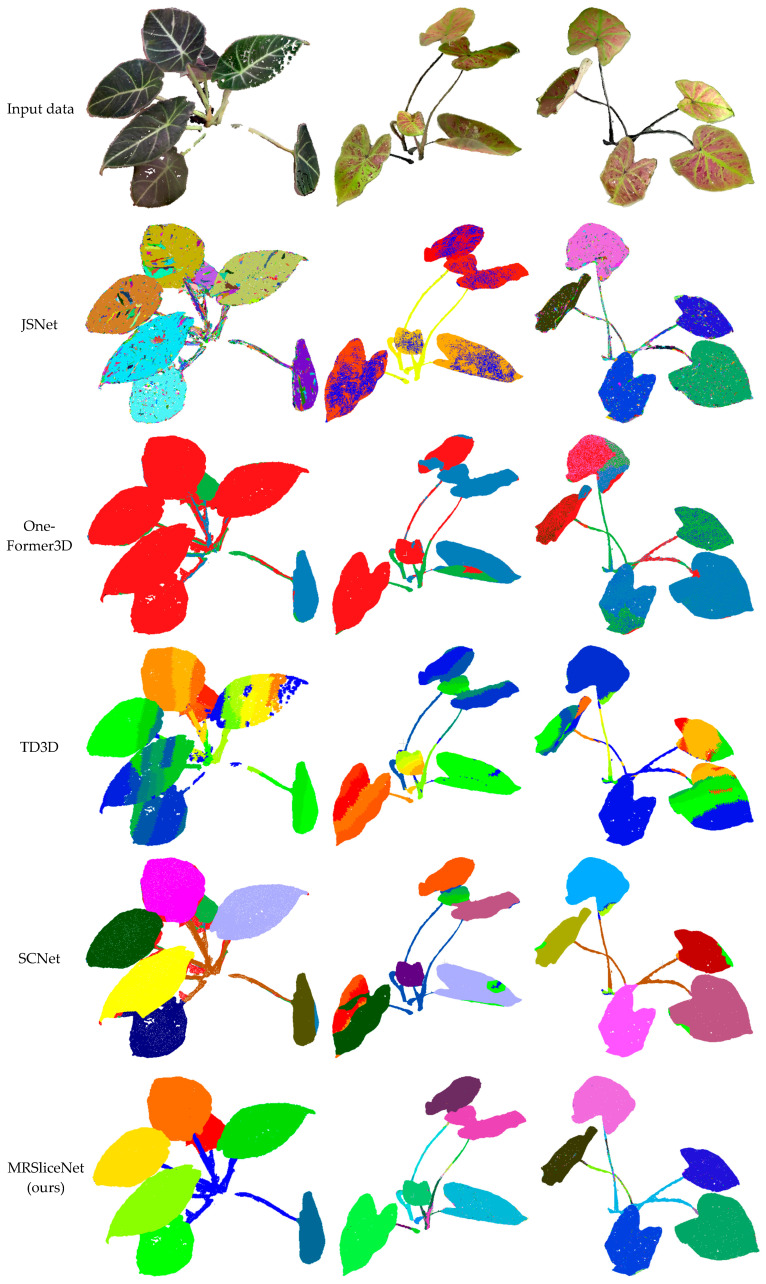
Segmentation results of the Dataset B plant point cloud. Different colors represent different instances.

We conducted a visual assessment of the instance segmentation performance using representative point cloud segments acquired from two potted plant datasets. The last rows of Figure 6 and Figure 7 present the qualitative prediction results containing distinctive foliage elements. The results demonstrate that the proposed method accurately assigns instance labels to the majority of points across diverse plant specimens within the test scenes. Segmentation outcomes for major plant components, such as stems and primary branches, are highly satisfactory—even in regions characterized by sparse point density or complex occlusions. Furthermore, the model effectively identifies smaller yet characteristic plant structures with limited instances, including individual leaves and petioles, indicating its capability to discern fine-grained botanical features. Particularly noteworthy is the method’s performance in leaf instance segmentation, where it successfully separates overlapping leaves and distinguishes adjacent foliage despite minimal geometric differentiation.

The framework maintains consistent segmentation quality across plants with varying leaf densities and arrangements, from sparse ornamental plants to dense foliage specimens. Critically, this robust performance is observed across the different species in our validation sets. For example, as shown in Figure 6, the model accurately segments both the large, simple leaves of a Philodendron and the smaller, compound leaves of a Schefflera. This demonstrates that MRSliceNet has learned to handle fundamental challenges like occlusion and boundary ambiguity that are common across many plant species, rather than memorizing species-specific features. The use of validation datasets comprising distinct species from those emphasized in training further evidences its generalization potential. In summary, the proposed segmentation framework performs robustly across all evaluated plant types and shows promising potential for generalization to a wider variety of botanical specimens, underscoring its practical value for digital plant phenotyping and automated horticultural management.

### 4.3. Quantitative Evaluation

Table 1 and Table 2 summarize the quantitative evaluation results of the proposed method for leaf instance segmentation on the two potted plant datasets, based on the metrics introduced in Section 4.1. A confusion matrix is provided to quantitatively illustrate the segmentation performance, both overall and per category, on the test regions. Globally, Dataset A achieves more favorable accuracy, with higher AP, AP_50_, and AP_25_ compared to Dataset B. This discrepancy stems largely from the higher morphological complexity of the specimens in Dataset B, where point clouds are characterized by severe leaf occlusion, intricate self-overlap, and higher density variations—all of which complicate accurate instance segmentation.

**Table 1:** Segmentation performance evaluation on dataset A. Note: AP denotes Average Precision. AP_25_ and AP_50_ are the AP values at IoU thresholds of 0.25 and 0.5, respectively.

Methods	AP (%)			AP_50_ (%)			AP_25_ (%)		
	Stem	Leaf	Overall	Stem	Leaf	Overall	Stem	Leaf	Overall
JSNet [61]	9.30	18.60	14.98	17.10	29.20	23.60	20.78	33.80	26.16
OneFormer3D [62]	25.98	28.26	27.08	31.68	39.60	35.59	40.02	51.80	46.05
TD3D [63]	14.41	20.49	17.45	22.58	27.32	24.95	32.93	34.44	33.68
SCNet [56]	46.75	50.63	48.95	55,46	62.81	60.56	60.12	67.85	64.06
MRSliceNet (ours)	50.80	58.94	55.04	60.95	69.48	65.37	69.00	78.56	74.68

**Table 2:** Segmentation performance evaluation on dataset B. Note: AP denotes Average Precision. AP_25_ and AP_50_ are the AP values at IoU thresholds of 0.25 and 0.5, respectively.

Methods	AP (%)			AP_50_ (%)			AP_25_ (%)		
	Stem	Leaf	Overall	Stem	Leaf	Overall	Stem	Leaf	Overall
JSNet [61]	8.77	10.90	9.40	15.85	29.03	22.56	18.90	35.66	27.25
OneFormer3D [62]	27.74	30.69	29.52	33.44	39.31	36.93	40.77	45.70	42.73
TD3D [63]	12.62	20.50	17.56	20.05	27.34	23.19	29.51	34.47	32.49
SCNet [56]	49.23	52.33	51.47	52.15	55.69	54.09	59.50	69.18	64.83
MRSliceNet (ours)	51.12	55.08	53.78	62.89	65.76	64.00	70.13	76.79	73.45

In terms of leaf instance segmentation performance, the proposed method achieves an AP of 55.04% ± 2.1%, AP_50_ of 65.37% ± 1.5%, and AP_25_ of 74.68% ± 1.2% on Dataset A. On Dataset B, it achieves an AP of 53.78% ± 2.4%, AP_50_ of 64.00% ± 1.7%, and AP_25_ of 73.45% ± 1.3%. The low standard deviations associated with these results indicate consistent performance across the diverse plant specimens in both datasets. This demonstrates the method’s robust capability to accurately delineate individual leaf instances even under challenging conditions such as dense foliage and overlapping structures. Qualitative analyses further confirm that the method effectively handles boundary ambiguity and occlusions, contributing to reliable instance-level recognition.

Nevertheless, the proposed method consistently delivers robust performance across both datasets. The proposed MRSliceNet effectively balances accuracy under different IoU thresholds, confirming its strong suitability for fine-grained instance segmentation of potted plant point clouds, particularly in the task of leaf instance segmentation.

### 4.4. Architecture Design Analysis

In the ablation study on the instance segmentation network for potted plant point clouds, we systematically evaluated the contributions of the three proposed core modules—the MRSM, the CFM, and the IACH—to the final segmentation performance. The specific experimental results are summarized in Table 3.

Initially, using only a basic point cloud encoder network (e.g., PointNet++) without any proposed modules, the model (i.e., Model A) performed poorly on dense and adherent leaf canopies, achieving only AP of 47.55%, AP_50_ of 57.02%, and AP_25_ of 64.77% in instance segmentation. The model struggled to distinguish between adjacent leaves, particularly under severe occlusion and ambiguous leaf boundaries. Introducing the MRSM module (i.e., Model B) increased the AP to 49.22%, AP_50_ to 59.03%, and AP_25_ to 68.73%. This module effectively captured discriminative local features, such as leaf margins and venation patterns, through its multi-scale slicing strategy, significantly improving boundary segmentation accuracy, especially in regions rich in fine details. However, without global semantic integration, some heavily adherent leaves were still incorrectly merged.

Further incorporating the CFM module (i.e., Model C) led to a substantial performance gain, raising the AP to 52.78%, AP_50_ to 62.05%, and AP_25_ to 70.95%. By leveraging self-attention mechanisms and feature pyramid fusion, the CFM integrated local features with global contextual information. This enabled each point to perceive not only its local geometry but also its semantic role and positional context within the overall plant structure, markedly alleviating issues of leaf adhesion and misclassification.

Finally, with the addition of the IACH module for discriminative embedding learning, the model achieved notable performance. To precisely quantify the impact of the semantic filtering mechanism within IACH, we introduce an intermediate model, Model D-NSF (No Semantic Filtering). This model uses the full IACH but does not filter out non-leaf points. It achieves an AP of 53.01%, AP_50_ of 63.35%, and AP_25_ of 72.19%. While it shows a clear improvement over Model C, confirming the effectiveness of the embedding learning itself, its performance is consistently lower than the full model. In contrast, the complete model (i.e., Model D), which includes semantic filtering, achieved the best performance with an AP of 53.78%, AP_50_ of 64.00%, and AP_25_ of 73.45% on the test set. The IACH optimized the embedding space by pulling points from the same leaf together while pushing apart those from different leaves. The quantitative comparison between Model D-NSF and Model D demonstrates that the semantic filtering mechanism, by excluding interference from stems and other non-leaf structures, is crucial for further enhancing both the purity and robustness of the final instance segmentation.

In conclusion, the ablation study confirms the complementary roles and necessity of the MRSM, CFM, and IACH modules. Furthermore, it quantitatively validates the specific contribution of the semantic filtering step within the IACH. They progressively enhance the overall network performance by addressing three critical aspects: local feature extraction, global semantic fusion, and instance-level discrimination, respectively.

## 5. Experimental Analysis and Discussion

The proposed method, MRSliceNet, is rigorously compared with other state-of-the-art point cloud instance segmentation approaches to further validate its effectiveness. Although our comparison includes representative methods such as JSNet [61], OneFormer3D [62], TD3D [63], and SCNet [56], the fast-paced evolution of 3D computer vision has prevented the inclusion of certain recent approaches—such as Transformer-based models like Mask3D [58]—due to challenges in achieving reproducible implementation within our specific experimental framework. Future work will incorporate comparisons with such emerging paradigms as their codebases and models mature [64]. To ensure a fair comparison, all selected methods were re-implemented and trained on our plant point cloud dataset using identical experimental configurations.

Quantitative comparison results, summarized in Table 1 and Table 2, demonstrate that our method achieves the best performance across all evaluation metrics, showcasing its robustness and superiority for leaf instance segmentation tasks. Specifically, our approach attained the highest scores and outperformed SCNet [56] (the second-best method) by significant margins of approximately 7%/2% in AP, 5%/10% in AP_50_, and 10%/9% in AP_25_. Meanwhile, JSNet [61] performed slightly worse than TD3D [63], with gaps of approximately 3%/8% in AP, 1%/1% in AP_50_, and 7%/5% in AP_25_. It is particularly noteworthy that our method achieves competitive results across most object categories while demonstrating exceptional performance in leaf instance segmentation. Our approach achieved the highest AP scores in segmenting challenging leaf categories (including “overlapped leaves”, “small leaves”, and “occluded leaves”) with AP values reaching 58.94% and 55.08% respectively, on two different plant point cloud datasets.

Visual comparisons in Figure 6 and Figure 7 further validate these findings, using three representative point clouds collected from different plant species. The results indicate that both SCNet [56] and our method can more accurately segment individual leaf instances, particularly in dense foliage regions. However, most comparison methods struggle with misclassifying points belonging to adjacent leaves, especially in areas with severe leaf occlusion and adhesion. The superior performance of our method can be attributed to its effective handling of scale variation in plant structures through a novel multi-scale feature learning and fusion scheme. For deep neural networks processing point clouds directly, capturing multi-scale information is crucial for accurate instance segmentation, particularly for plant-related objects that exhibit significant scale variations. Conventional sampling strategies often fail to preserve fine geometric details of small leaves while simultaneously maintaining global contextual information for larger structures.

We interpret this superior performance as a direct consequence of the synergistic effect of our multi-view recursive slicing and context fusion mechanisms. Unlike methods that struggle with complex 3D occlusions, our recursive slicing strategy effectively disassembles this problem into a series of more manageable 2D segmentation tasks across multiple planes. This innovation allows the network to isolate individual leaves in cross-section, proving particularly effective at separating densely packed and overlapping foliage. Furthermore, the context fusion module is critical for resolving boundary ambiguity; it aggregates information from adjacent slices and different feature scales, enabling the model to leverage both local details and global structural context to infer complete and accurate leaf boundaries.

These technical advancements have direct and meaningful biological relevance. Accurate instance segmentation is the foundational prerequisite for reliable, high-throughput phenotyping. The precise delineation of each leaf instance provided by our method directly translates into more accurate extraction of key morphological traits. For instance, it enables the computation of individual leaf area with high fidelity, a critical metric for studying plant growth and biomass accumulation. Moreover, by reconstructing the segmented instances in 3D, one can derive accurate measurements of leaf angle and azimuth, which are vital for understanding light interception efficiency and canopy architecture [65]. This level of accuracy, achieved under challenging real-world conditions of occlusion, marks a significant step towards automating the detailed analysis of plant morphology for both research and breeding applications.

Looking forward, while this work focuses on 3D structural segmentation, a promising and exciting future direction lies in its integration with other imaging modalities. Combining our detailed morphological data with physiological information obtained from techniques such as hyperspectral imaging or chlorophyll fluorescence analysis [16,66] could provide a comprehensive insight into the structure-function relationship within plants. Such a multi-modal phenotyping system would not only quantify plant architecture but also simultaneously assess photosynthetic efficiency and physiological status, offering a holistic view of plant health and performance. The development of these integrated analytical frameworks will be crucial for unlocking the full commercial and scientific potential of next-generation automated plant phenotyping systems.

## 6. Conclusions

In conclusion, this study successfully addresses the significant challenge of automatically segmenting individual leaves from highly dense and occluded point clouds of potted plants. We hypothesized that its integrated design—combining multi-scale recursive slicing, context fusion, and instance-aware clustering—would achieve superior segmentation performance by more effectively handling occlusion, geometric similarity, and uneven point density compared to existing methods. Our extensive experimental validation conclusively confirms this hypothesis. The proposed MRSliceNet framework realizes this design through the synergistic integration of three core modules: the MRSM for focused extraction of fine-grained local geometric features, the CFM for effectively integrating these local cues with global contextual information, and the IACH for generating highly discriminative embeddings. Experiments on multiple challenging datasets demonstrate that our method significantly outperforms existing state-of-the-art approaches in segmentation accuracy, producing results with clear boundaries and precise instance identification.

Looking forward, the MRSliceNet framework is not limited to the species studied here; its architecture is readily adaptable to a wide range of other plant species, enhancing its utility for both fundamental plant research and commercial phenotyping systems. Furthermore, a promising and powerful extension lies in the integration of our 3D point cloud analysis with complementary 2D or multispectral imaging techniques. Such a multi-modal system would be uniquely capable of simultaneously assessing plant structure, health, and physiological status, providing a holistic view of plant performance. We conclude by underlining that this strategic integration is anticipated to significantly increase the commercial and applied value of MRSliceNet, positioning it as a core component in the next generation of intelligent agricultural technology.

## Figures and Tables

**Table 3:** Ablation study performance evaluation on dataset B. Note: AP denotes Average Precision. AP_25_ and AP_50_ are the AP values at IoU thresholds of 0.25 and 0.5, respectively.

Methods	Moudle	AP (%)			AP_50_ (%)			AP_25_ (%)		
		Stem	Leaf	Overall	Stem	Leaf	Overall	Stem	Leaf	Overall
Model A	Baseline (PointNet++)	45.23	48.90	47.55	52.11	62.03	57.02	61.58	69.00	64.77
Model B	+MRSM	47.74	50.69	49.22	53.74	64.31	59.03	63.77	71.70	68.73
Model C	+MRSM + CFM	51.08	53.95	52.78	60.88	63.75	62.05	68.56	72.75	70.95
Model D-NSF	+MRSM + CFM + IACH (No SF)	51.11	54.60	53.01	61.25	65.00	63.35	69.15	75.03	72.19
Model D	+MRSM + CFM + IACH (Full)	51.12	55.08	53.78	62.89	65.76	64.00	70.13	76.79	73.45

## Data Availability

The data presented in this study are available on request from the corresponding author due to the large volume of the datasets and their specialized formats, which make them unsuitable for deposition in a public repository.
